# Linear-In-The-Parameters Oblique Least Squares (LOLS) Provides More Accurate Estimates of Density-Dependent Survival

**DOI:** 10.1371/journal.pone.0167418

**Published:** 2016-12-09

**Authors:** Vasco M. N. C. S. Vieira, Aschwin H. Engelen, Oscar R. Huanel, Marie-Laure Guillemin

**Affiliations:** 1 MARETEC, Instituto Superior Técnico, Universidade Técnica de Lisboa, Av. Rovisco Pais, Lisboa, Portugal; 2 CCMAR, Center of Marine Science,—CIMAR Laboratorio Associado, F.C.T., University of Algarve, Campus Gambelas, Faro, Portugal; 3 Instituto de Ciencias Ambientales y Evolutivas, Facultad de Ciencias, Universidad Austral de Chile, Casilla, Valdivia, Chile; 4 CNRS, Sorbonne Universités, UPMC University Paris VI, UMI 3614, Evolutionary Biology and Ecology of Algae, Station Biologique de Roscoff, CS, Place G. Tessier, Roscoff, France; Evergreen State College, UNITED STATES

## Abstract

Survival is a fundamental demographic component and the importance of its accurate estimation goes beyond the traditional estimation of life expectancy. The evolutionary stability of isomorphic biphasic life-cycles and the occurrence of its different ploidy phases at uneven abundances are hypothesized to be driven by differences in survival rates between haploids and diploids. We monitored *Gracilaria chilensis*, a commercially exploited red alga with an isomorphic biphasic life-cycle, having found density-dependent survival with competition and Allee effects. While estimating the linear-in-the-parameters survival function, all model I regression methods (i.e, vertical least squares) provided biased line-fits rendering them inappropriate for studies about ecology, evolution or population management. Hence, we developed an iterative two-step non-linear model II regression (i.e, oblique least squares), which provided improved line-fits and estimates of survival function parameters, while robust to the data aspects that usually turn the regression methods numerically unstable.

## Introduction

Survival is one of the determinants of population viability. Its accurate estimation and manipulation is essential for the management of endangered species, invasive species, plagues and commercially exploited species. Survival is also one of the fundamental drivers of fitness and life-cycle evolution. It has been hypothesized as one of the vital rates through which sympatric kelp species differentiate their adaptation to the environment and consequently, their niche occupation [1]. In isomorphic biphasic life-cycles, dissimilar survival rates between the haploid and diploid generations cause their uneven field abundances [2–6] and are necessary for the prevalence of this type of life-cycle [7]. Furthermore, when these species follow a life strategy dominated by investment in survival, it takes only small differences between haploid and diploid survival for niche differentiation [8–11]. Population density is one of the factors most conspicuously affecting survival. Its most generalized dynamics is decreasing survival with increasing densities due to competition for resources. It is the basic driver of the self-thinning of monospecific stands of plants [12–14], algae [15–18] and animals [19–20]. However, survival (as well as other vital rates) can also decrease at very low densities, a dynamics known as the Allee effects that occurs in animal, plant and algae populations by a multitude of factors [21,22].

The integration of both competition and Allee effects inevitably leads to a non-linear density-dependent survival function. To facilitate parameter estimation, this function can be transposed into a 3^rd^ degree polynomial in order to density (x). Although it is non-linear, if each of the x^n^ is considered as an independent variable, it becomes a linear-in-the-parameters polynomial of which coefficients can be estimated using Ordinary Least Squares (OLS) or other model I regression algorithms minimizing the vertical residuals. However, this class of methods is only valid when the error in the x estimates is minimal when compared to the error in the y estimates. The appropriate alternative is a Model II regression minimizing the residuals perpendicular to the regression line [23–27]. Unfortunately for the common user (i.e, non-specialized in numerical analysis), this class of methods requires complex calculus, particularly when applied to non-linear models. Furthermore, their numerical stability demands for sample sizes that are unrealistically large for most ecological applications. Hence, model II regressions are not frequently used in ecology and rarely so when non-linear models are involved.

In this study, we derived a density-dependent survival function for *Gracilaria chilensis*, a red seaweed with a classical isomorphic biphasic life cycle, and tested for differences among its haploid males, haploid females and diploids stages. We developed a non-linear model II regression with an improved line-fit even with small sample sizes. It is an iterative, two-step, Linear-in-the-parameters, Oblique Least Squares (LOLS) method, that is numerically stable in situations where other methods are usually unstable. To compare the parameters obtained from the several algorithms tested, we developed a user-friendly Matlab-based software package that includes a simple and short tutorial (S1 File).

## Gracilaria chilensis

This red alga is heavily cultivated and harvested for agar in the intertidal along the Chilean shore. Its life-cycle alternates between free living isomorphic haploid (gametophytes) and diploid (tetrasporophytes) phases. The gametophytes are either male or female [4,7,28]. Diploids (D), haploid males (M) and haploid females (F) were monitored in 5 intertidal rock-pools within 2 sites (Corral 39°52’27”S / 73°24’02” W and Niebla 39°55’47”S / 73°23’57”W) along the margins of the Valdivia river estuary, from October 2009 to February 2011 at 4 month intervals. No specific permissions were required for these locations and activities. The sampling sites were not in protected areas, *G*. *chilensis* is not an endangered or protected species, and the sampling method was non-destructive. All individuals within each rock-pool at each time were mapped. M, F and D fronds were identified using first the observation of reproductive structures in a tissue sample under a binocular microscope and the methodology developed in Guillemin et al. [29] for the remaining vegetative individuals. Frond length and diameter were recorded for each individual observed at each census in each rock pool. Individual biomass was represented by the volume (v_i_) of a cylinder of equal length and diameter as the frond, significantly correlated with dry weight (r^2^ = 0.877;*P* < 0.0001; *n* = 281). Every individual absent after 4 months was re-checked after 8 month for confirmation of death.

## The density-dependent survival (*s*)

**Fig 1:**
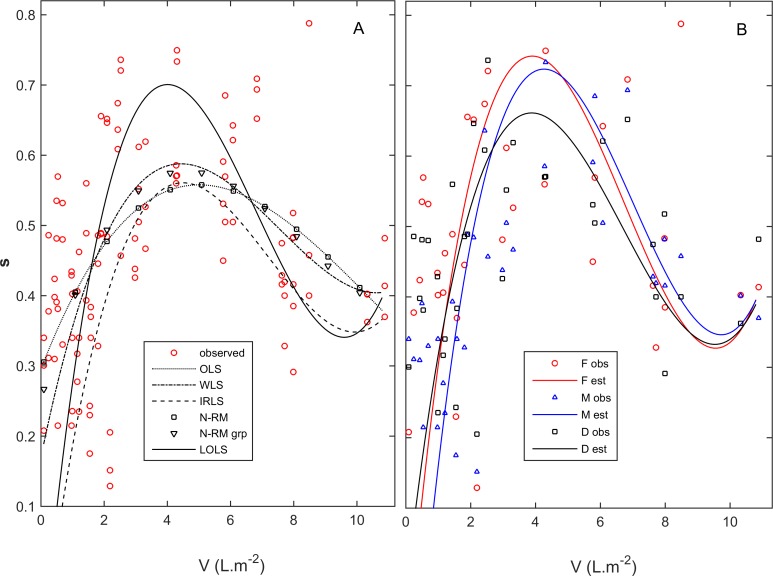
Linear-in-the-parameters regressions to estimate the survival (s) dependency on population density (V). A: all stages pooled together for an estimation by (OLS) Ordinary Least Squares, (WLS) Weigthed Least Squares, (IRLS) Iterative Re-weighted Least Squares, (N-RM) maximum likelihood estimation iterated by Newton-Raphson Method (grp) with data grouped, (LOLS) or Linear-in-the-parameters Oblique Least Squares. B: estimation by LOLS independently for (F) females, (M) males, and (D) diploids.

The biomass density of each pool was approximated by total frond volume per rock-pool area (V_p_ = ∑V_i_), rescaled to units of L∙m^-2^. The survival (*s)* dependency on *V*_*p*_ followed the shape of a quadratic function (Fig 1), with a peak survival at intermediate densities and increased mortality at lower and higher densities, corresponding to Allee effects and competition, respectively. The quadratic function *s* = *s*_*max*_*-b*_*0*_(*V*_*p*_-*V*_*opt*_)^2^ defined the maximum survival (*s*_*max*_) attained at an optimal biomass density (*V*_*opt*_). The parameter *b*_*0*_ represented the sensitivity of *s* to *V*_*p*_, with higher *b*_*0*_ yielding steeper curves. However, the sensitivity of *s* was observed to be asymmetrical relative to lower *vs* higher *V*_*p*_, meaning Allee effects and competition did not act proportionally. Therefore, *b*_*0*_ was replaced by *b*_*0*_(1+*b*_*1*_(*V*_*p*_-*V*_*opt*_)), resulting in a density-dependent survival function also including the sensitivity asymmetry parameter *b*_*1*_ (Eq 1). If survival changed more with higher densities (i.e, Allee effects < competition), then *b*_*1*_>0. On the other hand, if survival changed more with lower densities (i.e, Allee effects > competition), then *b*_*1*_<0.

$$s=s_{\max}-b_{0}\left(1+b_{1}(V_{p}-V_{\mathrm{opt}})\right)\cdot {\left(V_{p}-V_{\mathrm{opt}}\right)}^{2}$$ (1)

The procedure to estimate the parameters *s*_*max*_, *V*_*opt*_, *b*_*0*_ and *b*_*1*_ was to re-formulate Eq (1) as a 3^rd^ degree polynomial in order to *x* = *V*_*p*_ i.e, *s* = *β*_*0*_+*β*_*1*_*x*+*β*_*2*_*x*^2^+*β*_*3*_*x*^3^, and determine the *β*_*i*_ coefficients from a regression algorithm. The several algorithms tested are presented below in increasing order of complexity. Afterwards, the solutions to the survival parameters were obtained from Eq (2a). However, with *V*_*p*_ ranging from 0.1 to 12 and *s* ranging between 0.2 and 0.8, several methods had unstable calculus leading to unreliable results. Hence, both variables were forced to vary within {-1 1} by transforming *x* = (2*V*-^*max*^*V*-^*min*^*V*)/(^*max*^*V*-^*min*^*V*) and *y* = (2*s*-^*max*^s-^*min*^*s*)/(^*max*^s-^*min*^s), with the left superscripts ^*max*^ and ^*min*^ corresponding to the maximum and minimum observed values, respectively. While it solved the numerical instability, the estimation of the model parameters needed Eq (2b) proceeding Eq (2a).

$$\left\{ \begin{array}{c} x_{\mathrm{opt}}=\frac{\beta_{2}+\sqrt{\beta_{2}^{2}-3\beta_{1}\beta_{3}}}{-3\beta_{3}}\\ ṡ=\beta_{0}-\beta_{2}x_{\mathrm{opt}}^{2}-2\beta_{3}x_{\mathrm{opt}}^{3}\\ {\dot{b}}_{0}=-\beta_{2}-3\beta_{3}x_{\mathrm{opt}}\\ {\dot{b}}_{1}=\frac{\beta_{3}}{\beta_{2}+3\beta_{3}x_{\mathrm{opt}}} \end{array}\right.$$ (2a)

$$\left\{ \begin{array}{c} V_{\mathrm{opt}}=\frac{x_{\mathrm{opt}}(-)++}{2}\\ s_{\max}=\frac{{ṡ}_{\max}(-)++}{2}\\ b_{0}=\frac{2{\dot{b}}_{0}(-)}{{\left(-\right)}^{2}}\\ b_{1}=\frac{2{\dot{b}}_{1}}{-} \end{array}\right.$$ (2b)

### Ordinary Least Squares (OLS)

Polynomials of orders higher than 1 are non-linear models. However, considering the x^i^ as independent variables, polynomials are linear-in-the-parameters and these can be estimated by OLS. Calculus was simpler transposing the model to matrix algebra (Eq 3). Using matrix notation: **y** = **Xβ+ε**, where **y** was the (n×1) vector with the observations of y, **X** was the (n×4) matrix with the observations of x^i^ (i = 0,…,3), **β** was the (4×1) vector with the β_i_ coefficients and **ε** was the (n×1) vector with the error in the τ_p_ observations. Then, **β** was obtained from **β** = (**X**^T^**X**)^-1^**X**^T^**y**, where the superscript ^T^ denotes the matrix transpose.

$$\left[\begin{array}{c} y_{1}\\ \ldots \\ y_{n} \end{array}\right]=\left[\begin{array}{cccc} x_{1}^{0} & x_{1}^{1} & x_{1}^{2} & x_{1}^{3}\\ \ldots & \ldots & \ldots & \ldots \\ x_{n}^{0} & x_{n}^{1} & x_{n}^{2} & x_{n}^{3} \end{array}\right]\cdot \left[\begin{array}{c} \beta_{0}\\ \beta_{1}\\ \beta_{2}\\ \beta_{3} \end{array}\right]+\left[\begin{array}{c} \varepsilon_{1}\\ \ldots \\ \varepsilon_{n} \end{array}\right]$$ (3)

### Weigthed Least Squares (WLS)

The WLS estimated **β** weighting the observations by the (p×p) matrix **W**, the inverse of their variance-covariance matrix. Hence, the regression model was upgraded from the previous OLS to become **β** = (**X**^T^**WX**)^-1^**X**^T^**Wy**.

### Iterative Re-weigthed Least Squares (IRLS)

The IRLS estimated **β** minimizing the weighted y-residuals (Eq 4) while re-estimating their weights (w_p_) at each iteration (Eq 5). This procedure demanded for initial estimates to be provided. The interval 0<ϕ<2 guaranteed that the observations with smaller residuals i.e, closer to the central tendency, had iteratively increasing weight on the parameter estimation.

$$\beta_{t+1}=\underset{\beta }{\mathrm{argmin}}\sum_{p=1}^{n}w_{p}\beta_{t}{\left|y-c_{0}-c_{1}x-c_{2}x^{2}-c_{3}x^{3}\right|}^{\phi }$$ (4)

$$w_{p}={\left|y_{p}-c_{0}-c_{1}x-c_{2}x^{2}-c_{3}x^{3}\right|}^{\phi -2}$$ (5)

### Maximum Likelihood Estimation (MLE)

A preliminary manual fit suggested the error in the *s* estimates (*ε*_*p*_ = *y*_*obs*_-*y*_*est*_) followed a normal distribution *N*(0,σ^2^). Therefore, its associated likelihood function (ℒ) used the normal distribution formula (Eq 6). However, it was preferable to use its log-likelihood function (ℓ = logℒ) because a posterior step required the partial derivatives and these were easier to estimate from a sum (∑) than from a product (∏) and an exponent. The logarithmic function being monotonic, the maximum of ℒ and ℓ coincided. Hence, the estimates of **β** maximized ℓ(0,σ^2^|*ε*_*p*_) from Eqs (7) and (8) knowing that *ε*_*p*_ = f(*y*_*obs*,_ *y*_*est*_) and *y*_*est*_ = g(**β**,x).

$$L(0,\sigma^{2}\vee \varepsilon_{p})=\prod_{p=1}^{n}\frac{1}{\sqrt{2\pi \sigma^{2}}}exp\left[\frac{-\varepsilon_{p}^{2}}{2\sigma^{2}}\right]$$ (6)

$$l(0,\sigma^{2}|\varepsilon_{p})=\frac{-n}{2}\log(2{\pi \sigma }^{2})-\frac{1}{2\sigma^{2}}\sum_{p=1}^{n}{\left(y_{\mathrm{obs}}-\beta_{0}-\beta_{1}x-\beta_{2}x^{2}-\beta_{3}x^{3}\right)}^{2}$$ (7)

$$\left(\beta_{0},\beta_{1},\beta_{2},\beta_{3})=\underset{\beta }{\mathrm{argmax}}l(0,\sigma^{2}|\varepsilon_{p}\right)$$ (8)

When the maximum of ℓ was attained, the first-order partial derivatives of ℓ in order to **β** (i.e, ∂ℓ/∂β_i_) became zero. The Newton-Raphson method [30,31] numerically determined these roots from **β**_t+1_ = **β**_t_-f'(ℓ)/f''(ℓ). This was a system of 4 equations (∂ℓ/∂β_0_ = 0, ∂ℓ/∂β_1_ = 0, ∂ℓ/∂β_2_ = 0 and ∂ℓ/∂β_3_ = 0) with 4 unknowns (β_0_, β_1_, β_2_ and β_3_). However, σ^2^ was also unknown and needed to be estimated apart from ℓ as it was its forcing function. Then, **β** assumed the values that maximized ℓ (Eq 8) while minimizing σ^2^ (Eq 9). This duality corresponded to a system of 8 equations, where the four ∂σ^2^/∂β_i_ = 0 were added to the four ∂ℓ/∂β_i_ = 0 while preserving the four β_i_ unknowns. Finding its roots required adapting the Newton-Raphson method to Eq (10), with the (8×1) vector **u** of first order partial derivatives (Eq 11a), and the (8×4) Jacobian matrix **J** extracted from **u** (Eq 11b).

$$(\beta_{0},\beta_{1},\beta_{2},\beta_{3})=\underset{\beta }{\mathrm{argmin}}\sigma^{2}|y_{obs},x$$ (9)

$$\beta_{t+1}=\beta_{t}-{\left(J^{T}\cdot J\right)}^{-1}J^{T}\cdot u$$ (10)

$$u=\frac{\partial [\begin{array}{c} l\\ \sigma^{2} \end{array}]}{\partial \beta }=\left\{\begin{array}{c} \frac{\partial l}{\partial \beta_{0}}\\ \frac{\partial l}{\partial \beta_{1}}\\ \frac{\partial l}{\partial \beta_{2}}\\ \frac{\partial l}{\partial \beta_{3}}\\ \frac{\partial \sigma^{2}}{\partial \beta_{0}}\\ \frac{\partial \sigma^{2}}{\partial \beta_{1}}\\ \frac{\partial \sigma^{2}}{\partial \beta_{2}}\\ \frac{\partial \sigma^{2}}{\partial \beta_{3}} \end{array}\right\}$$ (11a)

$$J=\frac{\partial u}{\partial \beta }=\left\{\begin{array}{cccc} \frac{\partial^{2}l}{\partial \beta_{0}\partial \beta_{0}} & \frac{\partial^{2}l}{\partial \beta_{0}\partial \beta_{1}} & \frac{\partial^{2}l}{\partial \beta_{0}\partial \beta_{2}} & \frac{\partial^{2}l}{\partial \beta_{0}\partial \beta_{3}}\\ \frac{\partial^{2}l}{\partial \beta_{1}\partial \beta_{0}} & \frac{\partial^{2}l}{\partial \beta_{1}\partial \beta_{1}} & \frac{\partial^{2}l}{\partial \beta_{1}\partial \beta_{2}} & \frac{\partial^{2}l}{\partial \beta_{1}\partial \beta_{3}}\\ \frac{\partial^{2}l}{\partial \beta_{2}\partial \beta_{0}} & \frac{\partial^{2}l}{\partial \beta_{2}\partial \beta_{1}} & \frac{\partial^{2}l}{\partial \beta_{2}\partial \beta_{2}} & \frac{\partial^{2}l}{\partial \beta_{2}\partial \beta_{3}}\\ \frac{\partial^{2}l}{\partial \beta_{3}\partial \beta_{0}} & \frac{\partial^{2}l}{\partial \beta_{3}\partial \beta_{1}} & \frac{\partial^{2}l}{\partial \beta_{3}\partial \beta_{2}} & \frac{\partial^{2}l}{\partial \beta_{3}\partial \beta_{3}}\\ \frac{\partial^{2}\sigma^{2}}{\partial \beta_{0}\partial \beta_{0}} & \frac{\partial^{2}\sigma^{2}}{\partial \beta_{0}\partial \beta_{1}} & \frac{\partial^{2}\sigma^{2}}{\partial \beta_{0}\partial \beta_{2}} & \frac{\partial^{2}\sigma^{2}}{\partial \beta_{0}\partial \beta_{3}}\\ \frac{\partial^{2}\sigma^{2}}{\partial \beta_{1}\partial \beta_{0}} & \frac{\partial^{2}\sigma^{2}}{\partial \beta_{1}\partial \beta_{1}} & \frac{\partial^{2}\sigma^{2}}{\partial \beta_{1}\partial \beta_{2}} & \frac{\partial^{2}\sigma^{2}}{\partial \beta_{1}\partial \beta_{3}}\\ \frac{\partial^{2}\sigma^{2}}{\partial \beta_{2}\partial \beta_{0}} & \frac{\partial^{2}\sigma^{2}}{\partial \beta_{2}\partial \beta_{1}} & \frac{\partial^{2}\sigma^{2}}{\partial \beta_{2}\partial \beta_{2}} & \frac{\partial^{2}\sigma^{2}}{\partial \beta_{2}\partial \beta_{3}}\\ \frac{\partial^{2}\sigma^{2}}{\partial \beta_{3}\partial \beta_{0}} & \frac{\partial^{2}\sigma^{2}}{\partial \beta_{3}\partial \beta_{1}} & \frac{\partial^{2}\sigma^{2}}{\partial \beta_{3}\partial \beta_{2}} & \frac{\partial^{2}\sigma^{2}}{\partial \beta_{3}\partial \beta_{3}} \end{array}\right\}$$ (11b)

The computation of **u** and **J** was easier with a calculus vectorization similar to that by Vieira et al. [32]. It started by defining **M** (Eq 12) with entries *μ*_*m*,*k*_ = ∑*x*^m^∙*s*_*ob*s_^k^, · m = 0,…,6 ∧ k = 0,…,2. Matrix algebra estimated faster *μ* = {*x*^0^, *x*^1^, *x*^2^, *x*^3^, *x*^4^, *x*^5^, *x*^6^}^T^∙{*y*^0^, *y*^1^, *y*^2^}. It was also defined **В** = {1, -*β*_*0*_, -*β*_*1*_, -*β*_*2*_, -*β*_*3*_}. This way ℓ could be estimated from Eq (13) and σ^2^ from Eq (14), were n is the sample size and ⊗ is the Hadamard product i.e, matrix element-wise.

$$M=\left[\begin{array}{ccccc} \mu_{0,2} & \mu_{0,1} & \mu_{1,1} & \mu_{2,1} & \mu_{3,1}\\ \mu_{0,1} & \mu_{0,0} & \mu_{1,0} & \mu_{2,0} & \mu_{3,0}\\ \mu_{1,1} & \mu_{1,0} & \mu_{2,0} & \mu_{3,0} & \mu_{4,0}\\ \mu_{2,1} & \mu_{2,0} & \mu_{3,0} & \mu_{4,0} & \mu_{5,0}\\ \mu_{3,1} & \mu_{3,0} & \mu_{4,0} & \mu_{5,0} & \mu_{6,0} \end{array}\right]$$ (12)

$$l(0,\sigma^{2}\vee \varepsilon_{p})=\frac{-n}{2}\log(2\pi \sigma^{2})-\frac{1}{2\sigma^{2}}\sum \left({Β}^{T}\cdot Β⊗M\right)$$ (13)

$$\sigma^{2}=\frac{1}{n}\sum \left({Β}^{T}\cdot Β⊗M\right)$$ (14)

Their partial derivatives had the vectorial representation v_i_ = ∂**В**/∂c_i_, corresponding to **v**_**0**_ = {0, -1, 0, 0, 0}, **v**_**1**_ = {0, 0, -1, 0, 0}, **v**_**2**_ = {0, 0, 0, -1, 0}and **v**_**3**_ = {0, 0, 0, 0, -1}. Then, **u** and **J** were estimated inserting Eqs (15) to (18) into Eqs (11a and 11b).

$$\frac{\partial l}{\partial \beta_{i}}=\frac{-1}{2\sigma^{2}}\sum \left(v_{i}^{T}\cdot Β⊗M+{Β}^{T}\cdot v_{i}⊗M\right);\;\forall i=0,\ldots 3$$ (15)

$$\frac{\partial \sigma^{2}}{\partial \beta_{i}}=\frac{1}{n}\sum \left(v_{i}^{T}\cdot Β⊗M+{Β}^{T}\cdot v_{i}⊗M\right);\;\forall i=0,\ldots 3$$ (16)

$$\frac{\partial^{2}l}{\partial \beta_{i}\partial \beta_{j}}=\frac{-1}{2\sigma^{2}}\sum \left(v_{i}^{T}\cdot v_{j}⊗M+v_{j}^{T}\cdot v_{i}⊗M\right);\;\forall i=0,\ldots 3$$ (17)

$$\frac{\partial \sigma^{2}}{\partial \beta_{i}\partial \beta_{j}}=\frac{1}{n}\sum \left(v_{i}^{T}\cdot v_{j}⊗M+v_{j}^{T}\cdot v_{i}⊗M\right);\;\forall i=0,\ldots 3$$ (18)

The Newton-Raphson method is very sensitive and prone to overshooting the roots estimates, and so requires initial conditions that are not too far from the solution. Hence, we preformed a manual line-fit to estimate a provisional **β** by solving the systems of Eqs (2a) and (2b) backwards.

The method was improved by grouping the data into three V classes: (A) 0<V<4, (B) 4<V<5 and (C) 5<V<12 L∙m^-2^, with class B giving special attention to the line-fit in the area where *s*_*max*_ and *V*_*opt*_ occurred. The MLE was adapted to run simultaneously over the three classes by concatenating {ℓ_A_ σ^2^_A_ ℓ_B_ σ^2^_B_ ℓ_C_ σ^2^_C_}^T^. The system changed to 24 equations with 4 unknowns, with roots determined from Eqs (10) and (11), where **u** was a (24×1) vector and **J** a (24×4) matrix. Eqs (13) to (18) were solved independently for groups A, B and C. An analysis to the line-fit evolution during the Newton-Raphson iterations demonstrated that σ^2^_A_ had a far greater weight than σ^2^_B_ or σ^2^_C_, with its optimization even driving C into a worsening line-fit. To overcome this bias, the optimization of σ^2^ was standardized to its value at the previous iteration, which only required replacing n by nσ^2^ in the denominators of Eqs (16) and (18).

### Linear-in-the-parameters Oblique Least Squares (LOLS)

This is a model II regression minimizing the oblique residuals (i.e, perpendicular to the regression line). However, because the line was curved, each iteration comprised a first step to determine the vectors perpendicular to the third degree polynomial and a second step minimizing the sum of their magnitudes.

Step 1: Each observation was defined by vector {*x*,*y*} and their estimated values {*ẋ*,*ẏ*}. In cartesian coordinates, each residual was defined as vector **A** = {*x*-*ẋ*,*y*-*ẏ*}. **A** perpendicular to the polynomial was determined by making it perpendicular to the polynomial's derivative, also defined in Cartesian coordinates as vector **B** = {δ,δ∙∂*ẏ*/∂*ẋ*}, by preference using small δ. When vectors **A** and **B** were perpendicular, their inner product was null i.e, **A**◦**B** = 0. Hence, *ẋ* and *ẏ* were determined nesting Eq (19).

$$\begin{array}{c} (x-\dot{x})\delta +(y-\dot{y})\delta \frac{\partial \dot{y}}{\partial \dot{x}}=0\\ \dot{y}=\beta_{0}+\beta_{1}\dot{x}+\beta_{2}{\dot{x}}^{2}+\beta_{3}{\dot{x}}^{3}\\ \frac{\partial \dot{y}}{\partial \dot{x}}=\beta_{1}+2\beta_{2}\dot{x}+3\beta_{3}{\dot{x}}^{2} \end{array}$$ (19)

The calculus was performed using matrix algebra. The vectors with the y components of **A** and **B** were defined as **A**_**y**_ = {*y*,-*β*_*0*_,-*β*_*1*_*ẋ*,-*β*_*2*_*ẋ*^2^,-*β*_*3*_*ẋ*^3^} and **B**_**y**_ = {*β*_*1*_,2*β*_*2*_*ẋ*,3*β*_*3*_*ẋ*^2^}. The inner product was re-written as:

$$A{}^{\circ}B=\delta (x-\dot{x})+\delta \sum \left(A_{y}^{T}\cdot B_{y}\right)$$ (20)

The Newton-Raphson method generally took four iterations of *ẋ*_t+1_ = *ẋ*_t_-f(*ẋ*)/f'(*ẋ*) to converge into the roots of Eq (20) with a change rate<0,1%. The derivative f'(*ẋ*) was given by Eq (21), which required the derivatives of **A**_**y**_ (Eq 22) and **B**_**y**_ (Eq 23).

$$f\prime (\dot{x})=-\delta +\delta \sum \left(\frac{\partial }{\partial \dot{x}}A_{y}^{T}\cdot B_{y}+A_{y}^{T}\cdot \frac{\partial }{\partial \dot{x}}B_{y}\right)$$ (21)

$$\frac{\partial }{\partial \dot{x}}A_{y}=\{0,0,-\beta_{1},-2\beta_{2}\dot{x},-3\beta_{3}{\dot{x}}^{2}\}$$ (22)

$$\frac{\partial }{\partial \dot{x}}B_{y}=\{0,2\beta_{2},6\beta_{3}\dot{x}\}$$ (23)

A provisional **β** was required, providing the initial conditions for the first time step 1 was implemented. This was arbitrarily chosen aiming to be close to the expected final solution. Subsequent re-runs of step 1 used the **β** provided by step 2.

Step 2: the magnitude of each oblique residual was given by |res| = ((*x*-*ẋ*)^2^+(*y*-**Ẋβ**)^2^)^1/2^, with the (1×4) design matrix **Ẋ** = {**1**, **ẋ, ẋ**^**2**^**, ẋ**^**3**^}. The minimum sum of their squares i.e, SSE = ∑res^2^, was achieved when ∂SSE/∂**β** = 0. These roots were found numerically by iterating the Newton-Rahpson method under the form **β**_t+1_ = **β**_t_-**SSE**'/**SSE**''. The numerator **SSE**' was the (4×1) vector with the first order partial derivatives ∂SSE/∂*β*_*i*_ = -∑(*y*-**Ẋβ**)ẋ^i^,∀ i = 0,…,3. Upgrading **Ẋ** to the (n×4) design matrix and *y* to the (n×1) vector **y** including all n observations, matrix calculus estimated **SSE**' = **Ẋ**^**T**^(**Ẋβ-y**). The denominator **SSE**'' was the (4×4) Jacobian matrix with the second order partial derivatives ∂^2^SSE/∂β_i_∂β_j_ = ∑*ẋ*^i^*ẋ*^j^, ∀ i,j = 0,…,3. Matrix calculus estimated it from **SSE**'' = (**Ẋ**^**T**^**Ẋ**). Merging all yielded the incremental **β**_t+1_ = **β**_t_-(**Ẋ**^**T**^**Ẋ**)^-1^**Ẋ**^**T**^(**Ẋβ-y**). Only a single iteration within step 1 was performed, after which the perpendicular coordinates of the residuals needed to be re-estimated.

### Testing differences between life-cycle stages

We tested whether the parameters *s*_*max*_, *V*_*opt*_, *b*_*0*_ and *b*_*1*_ changed significantly with ploidy or sex using non-parametric permutation tests with 1000 randomizations. The test accuracy was improved by keeping the identity of the observations relatively to the non-tested properties i.e, observations were permuted within their site and season [17,18]. A suited definition of the survival function required merging sites and seasons. Thus, differences between treatments within these factors could not be tested. The sample size was 35 and one degree of freedom was lost with the estimation of each of the four model parameters. Because *V*_*opt*_ was the only parameter whose estimation was independent, the ANCOVA approach was followed i.e, first we estimated whether *V*_*opt*_ was significantly different among ploidies or sexes. Only in case it was not, did we estimate whether *τ*_*max*_, *b*_*0*_ and *b*_*1*_ were significantly different among ploidies or sexes.

## Results and Discussion

*Gracilaria chilensis* exhibited a clear pattern of maximum survival attained at optimal intermediate densities (Fig 1). The decreased survival of the ramets at higher densities has been widely detected in algae and attributed to competition [15–18]. But their decreased survival at lower densities (Allee effects) has hardly been reported. Crowding has been demonstrated to protect from desiccation in intertidal algae stands [33] and we hypothesize that it may also protect against hydrodynamic stress. The LOLS applied to each of the life-cycle stages showed no significant differences between males, females and diploids for any of the survival function parameters (Fig 1B and Table 1). If there were any actual differences between the density-dependent survival of the stages, these differences were masked by the error. Whenever testing the relation between variables x and y, there are two usual sources of error: (i) measurement error, and (ii) the influence of extra variables over x and/or y. In our case, a weakness of the data set constituted a third source of error undermining the parameter estimation: our observations predominantly corresponded to low population densities and thus, the line-fits that described the dynamics at high population densities were relatively weak. Our four monthly survey interval enhanced this issue. In hind side, a shorter time interval would have provide better density-dependent survival data and a better definition of the full function.

**Table 1:** The ploidy specific survival functions with parameters estimated by the LOLS (value) and the significance (p) of their differences among (M) males, (F) females and (D) diploids, as estimated from randomization tests. Sample size n = 35.

	s_max_			V_opt_			b_0_			b_1_		
	M	F	D	M	F	D	M	F	D	M	F	D
(value)	.724	.742	.661	4.261	3.898	3.88	.038	.039	.031	-.122	-.118	-.118
(p)	M	F	D	M	F	D	M	F	D	M	F	D
M		.732	.358		.147	.121		.898	.514		.658	.662
F			.283			.932			.484			.992

Finding the best parameter estimates also required solving problems specific to the numerical methods (Fig 1A and Table 2). The OLS line-fit concentrated the positive residuals on the centre, largely underestimating the maximum survival rate (*s*_*max*_) at optimal densities. The WLS represented only a slight improvement, while the IRLS represent no improvement at all. Besides, when diploids, males and females were pooled separately (n = 3 months×3 years×5 pools = 35) the results varied significantly with the ϕ and the initial estimates provided. Upon smaller sample sizes, particular observations erroneously became anchor-points (or attractors) to the IRLS line-fit. The standard MLE provided a line-fit equal to the OLS. When the data was split into the three V classes, it became evident that the optimization of the fit at lower densities was worsening the fit at intermediate and higher densities. While σ^2^_A_ decreased from 335∙10^−4^ to 187∙10^−4^, σ^2^_B_ increased from 73∙10^−4^ to 155∙10^−4^ and σ^2^_C_ increased from 117∙10^−4^ to 123∙10^−4^. The MLE with these three V classes weighted by the inverse of their variance provided a line-fit that was only slightly different. All these methods fall upon the class of model I regressions, characterized by a minimization of the vertical residuals i.e, of the error in the response variable. Besides method-specific fails, their generalized bias also resulted from the fact that the error in the estimation of the predictor was larger than the error in the estimation of the response. In fact, we had more confidence in the estimation of the survival rates (ramets were either dead or alive) than in the estimation of the individual sizes and consequent population biomass densities. In such cases, model I regressions are inadequate and model II regression are the proper alternative [25–27]. Classical error-in-the-variables algorithms for model II regressions estimate the true parameter values by use of high-order moments (or cumulants). However, besides extremely complicated for non-linear models, the use of high-order moments turns them numerically unstable, requiring extremely large data sets (n>900) for reliable estimates [34–36]. The instrumental variables (an alternative class of error-in-the-variables algorithms) demands for a subset of data with known errors to train the algorithm into the estimation of the true parameter values. Principal Components Analysis (PCA) and Reduced Major Axis (RMA) are model II regression algorithms with a different strategy: they do not aim at estimating the true parameter values but simply at minimizing the oblique residuals i.e., perpendicular to the line-fit [23–27]. Although this simplifies and numerically stabilizes them, working fine with smaller data sets, they are exclusive to linear models, and thus inapplicable to our linear-in-the-parameters third degree polynomial.

**Table 2:** The overall survival function with parameters estimated by different methods. Sample size n = 105.

	s_max_	V_opt_	b_0_	b_1_
OLS	0.558	5.004	0.008	-0.059
WLS	0.588	4.432	0.014	-0.108
IRLS	0.561	4.489	0.021	-0.123
N-RM	0.558	5.004	0.008	-0.059
N-RM gp	0.578	4.786	0.011	-0.087
LOLS	0.701	4.009	0.035	-0.119

It was based in the PCA and RMA rationale that we developed the Linear-in-the-parameters Oblique Least Squares (LOLS) regression method, minimizing the squared residuals perpendicular to a curve line. This procedure distributed the positive and negative residuals evenly along the x axis, contrasting with the other tested algorithms (Fig 1A). However, as the LOLS had no closed form solution, it required being iterated over two steps: one to update the orientations of the vectors of residuals and another to minimize their magnitudes. This method was still fallible when (-if) working with the original variables. Whenever at least one of the variables ranged beyond |1| or their variances were conspicuously unbalanced, the method became numerically unstable. Often, the vertical (or the horizontal) component of the oblique residuals became meaningless and the LOLS was in practice performing a horizontal (or vertical) regression. This problem was solved by transforming both variables to range within -1<x_j_<1. Overall, although computationally more demanding, the LOLS was still fast providing a solution that was much more satisfactory than the solutions provided by the former algorithms (Fig 1A and Table 2). It estimated conspicuously higher maximum survival rate (*s*_*max*_) at lower optimum density (*V*_*opt*_) with higher density-dependence (*b*_*0*_) and bigger asymmetry between competition and Allee effects (*b*_*1*_). An apparent draw-back was the function increasing back again at the extreme of higher densities. However, this error was only a consequence of the small number of observations in that area of the function, it should not happen with more complete data sets and hence should be negligible for the majority of future model applications. The consistency of the LOLS fits among stages (n = 35) contrasted with the inconsistency demonstrated by other methods, and in particular by the IRLS.

## Conclusions

The accurate estimation of survival is essential for ecological and evolutionary studies, as well as for population management. As found in other organisms, the survival of *Gracilaria chilensis* is sensitive to both competition and Allee effects, and thus is optimized at intermediate densities. Although computationally more demanding, the LOLS algorithm was the method providing the best description of this non-linear function. This method is better suited in situations demanding for non-linear model II regression methods i.e, when the error in the estimation of the predictor *x* is comparable to the error in the estimation of the response *y*. Furthermore, its conjugation with the transformations of both predictor and response to vary within {-1, 1} made the LOLS robust in situations where the other methods tested were fallible and numerically unstable. Therefore, the LOLS should have a wide applicability.

## Supporting Information

S1 File S1 software and data.zip. Software package and *Gracilaria chilensis* data.
(ZIP)
