# Correction: Linear-In-The-Parameters Oblique Least Squares (LOLS) Provides More Accurate Estimates of Density-Dependent Survival

**DOI:** 10.1371/journal.pone.0174156

**Published:** 2017-03-13

**Authors:** Vasco M. N. C. S. Vieira, Aschwin H. Engelen, Oscar R. Huanel, Marie-Laure Guillemin

In the section titled “The density-dependent survival (*s*),” there is an error in equation (2b). Please see the correct equation (2b) here:

$$\{\begin{array}{c} V_{\text{opt}}=\frac{x_{\text{opt}}\left(V_{\text{max}}-V_{\text{min}}\right)+V_{\text{max}}+V_{\text{min}}}{2}\\ b_{0}=\frac{4{\dot{b}}_{0}}{{\left(V_{\text{max}}-V_{\text{min}}\right)}^{2}}\\ b_{1}=\frac{2{\dot{b}}_{1}}{V_{\text{max}}-V_{\text{min}}} \end{array}$$

In the section titled “Maximum Likelihood Estimation (MLE),” there are errors in equation (6) and equation (13). Please see the correct equation (6) and equation (13) here:

$$L\left(0,\sigma^{2}|\varepsilon_{\text{p}}\right)=\overset{n}{\underset{p=1}{\prod }}\frac{1}{\sqrt{2\pi \sigma^{2}}}exp\left[\frac{-\varepsilon_{p}^{2}}{2\sigma^{2}}\right]$$

$$l\left(0,\sigma^{2}|\varepsilon_{\text{p}}\right)=\frac{-n}{2}\text{log}\left(2\pi \sigma^{2}\right)-\frac{1}{2\sigma^{2}}\sum \left(B^{T}\cdot B⊗M\right)$$
