# Supplementary material for: Linear-In-The-Parameters Oblique Least Squares (LOLS) Provides More Accurate Estimates of Density-Dependent Survival
Source: PLoS One. 2016 Dec 9;11(12):e0167418. doi: 10.1371/journal.pone.0167418 (PMC5147871; doi:10.1371/journal.pone.0167418)
Supplement: S1 File — Software package and Gracilaria chilensis data. (ZIP) [file pone.0167418.s001.zip › Tutorial.doc]

TUTORIAL

The data and software run in Matlab ®. The **Data.mat** file has the data in a cell array called ‘datatable’. This is a table where the columns 1-4 have independent categorical variables. In this case it is ‘year’, ‘month’, ‘site’ and ploidy. Column 5 has the density given as total volume (x variable) while column 6 has density in total volume minus the volume of the bigger individual. This aims at eliminating bias from abnormally large individuals.

The **survival_single_fit.m** file executes a single line-fit whereas the s**urvival_multiple_fit.m** file compares among the life-cycle stages. The ‘Settings’ section is where the user edits how he wishes to do his analysis. The ‘Calculus’ section is not to be edited by the user.

The results are provided in the data structure ‘Results’. The estimated parameters are given in ‘Results.stage’ while the significances of differences between stages are given in ‘Results.sig_dif’.

The ‘OptEvol’ cell array gives the rate of convergence of the parameters estimation from time t to t+1. The time series is in the 1stdimension, the parameters are in the 2^nd^ dimension and the stages in the 3^rd^ dimension.
